# Interstitial pneumonia in a patient treated with TAS-102 for metastatic colorectal cancer: a case report

**DOI:** 10.1186/s13256-016-1097-y

**Published:** 2016-11-03

**Authors:** Hideki Kamei, Nobuya Ishibashi, Masahiko Tanigawa, Keizo Yamaguchi, Masafumi Uchida, Yoshito Akagi

**Affiliations:** 1 Department of Surgery, Kurume University School of Medicine, 67 Asahimachi, Kurume, Fukuoka 830-0011 Japan; 2 Department of Radiology, Kurume University School of Medicine, 67 Asahimachi, Kurume, Fukuoka 830-0011 Japan

**Keywords:** TAS-102, Colorectal cancer, Interstitial pneumonia

## Abstract

**Background:**

TAS-102, a new treatment option for patients with metastatic colorectal cancer that is refractory or intolerant to standard therapies, has been improving survival with acceptable tolerability and adverse events. Adverse hematological events associated with TAS-102 treatment were extensively profiled in the RECOURSE trial, but pulmonary toxicities associated with TAS-102 therapy are distinctly uncommon. In a recent early post-marketing phase vigilance on TAS-102 in Japan, seven cases of pulmonary disease were reported, but patient follow-up in this study was incomplete. Here, we present the first case of interstitial lung disease occurring in association with TAS-102 treatment.

**Case presentation:**

A 57-year-old Japanese man who had previously received two standard treatments was admitted in 2014, at which time we administered TAS-102 (110 mg/day) as a third-line chemotherapy. He was safely treated with TAS-102 for the first planned cycle; however, approximately 4 days after receiving the second cycle of TAS-102, he complained of high fever and subsequent dyspnea with severe hypoxemia and went to the emergency room. A chest X-ray revealed diffuse coarse reticular shadows with ground-glass opacity on both lungs. Furthermore, a chest computed tomography scan showed thickening of the bronchovascular bundles with extensive ground-glass opacification and pleural effusions in both lung fields.

In addition, a peripheral blood lymphocyte stimulation test with TAS-102 showed higher values compared with control samples. Consequently, we suspected drug-induced interstitial pneumonia, and discontinued treatment. Our patient was given an initial administration of high-dose methylprednisolone (1000 mg/day) for 3 days and oxygen. Our patient was discharged with oral prednisolone (20 mg/day) and improved symptomatically and radiologically.

**Conclusions:**

These findings suggest that interstitial pneumonia is a rare complication of TAS-102 chemotherapy, but the possibility of interstitial pneumonia should always be considered when a patient presents with a respiratory disorder while undergoing TAS-102 systemic chemotherapy.

Prompt discontinuation of TAS-102 and treatment with high-dosage corticosteroids is needed to avoid exacerbating respiratory symptoms.

## Background

TAS-102 was first approved in Japan in March 2014 for the treatment of metastatic colorectal cancer (mCRC) and has demonstrated efficacy in fluorouracil (5-Fu) refractory patients based on having a different mechanism of action [1]. The safety of TAS-102 with the Japanese recommended dose has also been independently confirmed [2]. The toxicity profile of TAS-102 differs from the known adverse effects of 5-Fu and its derivatives. In the RECOURSE trial, TAS-102 was associated with a favorable overall safety profile, but treatment was accompanied with a significant increase of hematological toxicities. In particular, 38 % of patients presented with grade 3–4 neutropenia, although febrile neutropenia was only observed in 4 % of the patients [3]. However, TAS-102 has never been clearly associated with incidences of interstitial pneumonia (IP). According to the phase 2 trial (J003 trial) involving 169 Japanese patients, there was one suspected case of interstitial lung disease (ILD) [4]. A few reports of IP were reported for TAS-102 in combined therapy with fluorouracil, leucovorin, and oxaliplatin (FOLFOX4) [0.6 % (2 out of 322 patients)] and with fluorouracil, leucovorin, and irinotecan (FOLFIRI) [0.7 % (2 out of 302 patients)] [5]. Induced ILD has also been reported in 1.3 % (39 out of 3085) of patients receiving the molecularly targeted therapy panitumumab [6]. There have been no reports of ILD in patients receiving TAS-102 chemotherapy, whereas seven cases were reported from the early post-marketing phase vigilance (EPPV) on TAS-102 in Japan [7]. Herein, to the best of our knowledge, we report the first case of IP associated with TAS-102 therapy in a patient with mCRC.

## Case presentation

The patient was a 57-year-old Japanese man with rectal adenocarcinoma. The tumor was located in the middle rectum and had invaded the serosa with regional metastases. His past medical history was hypertension, hepatitis C virus infection, subarachnoid hemorrhage, and chronic obstructive pulmonary disease. His social history was significant, including a 20 pack-year smoking history. A low anterior resection was performed in 2010; pathology revealed a stage IIIB moderately differentiated, *KRAS* wild-type adenocarcinoma, with one out of 15 lymph nodes testing positive. After surgery, he received 12 cycles of adjuvant FOLFOX chemotherapy. One year later, a subsequent positron emission tomography/computed tomography (PET/CT) scan showed evidence of recurrence, with liver, lung and pelvic lymph node metastases. Additionally, his carcinoembryonic antigen (CEA) values had increased to 23 ng/ml. He subsequently received 15 cycles of FOLFIRI + cetuximab therapy followed by 12 cycles of FOLFOX + bevacizumab; however, a PET/CT scan in June 2014 revealed progressive disease.

**Fig. 1:**
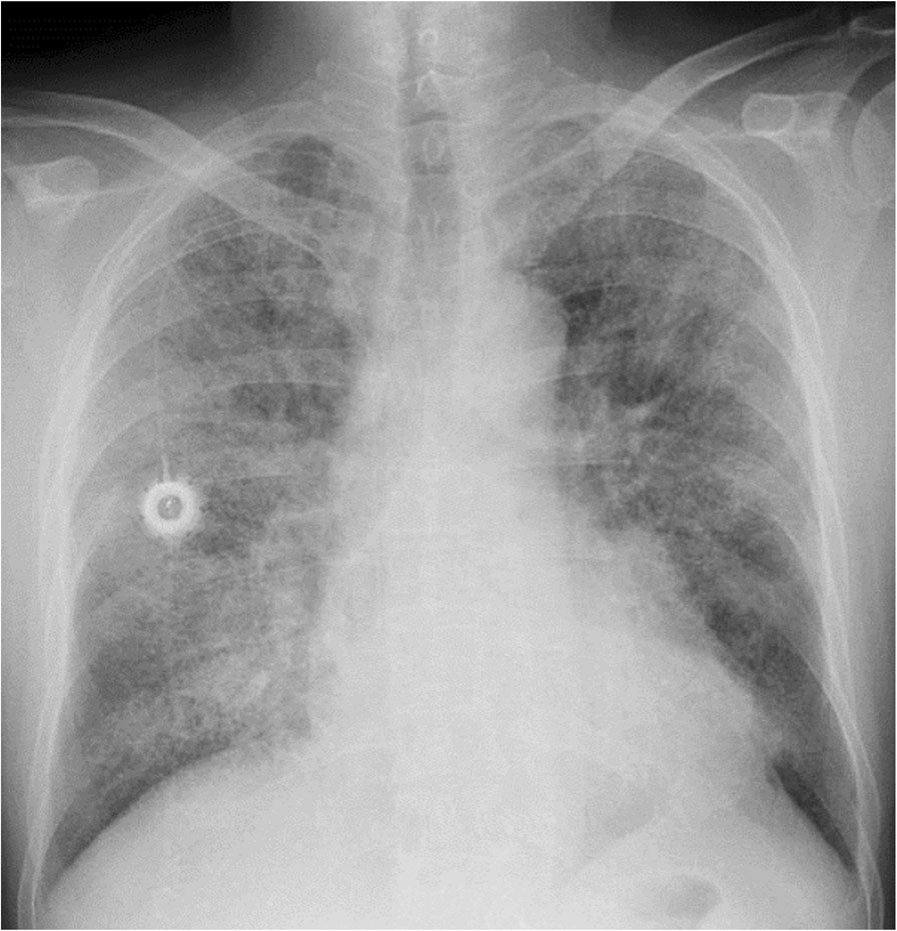
Chest X-ray obtained in June 2014 showing reticulonodular shadows in both lung fields

**Fig. 2:**
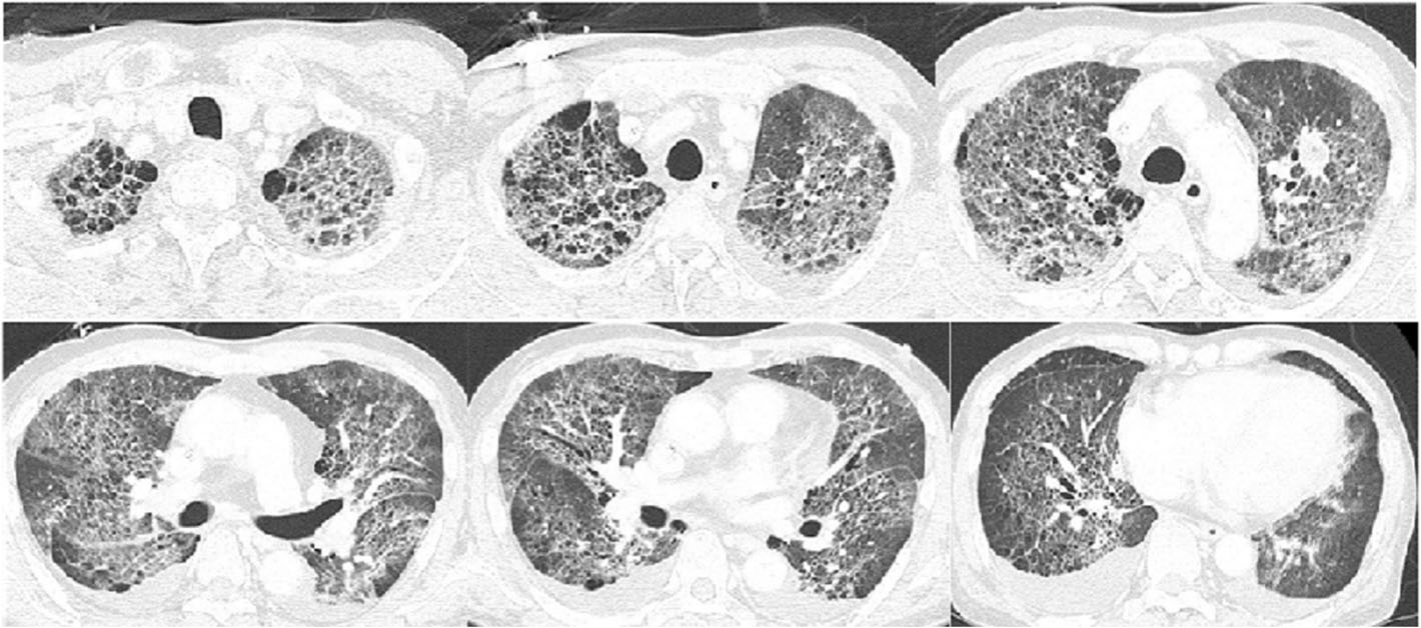
Computed tomography scan showing features of interstitial lung disease

TAS-102 was subsequently considered as a third-line chemotherapy option, and he began 110 mg daily TAS-102 treatment in June 2014. He had no history of food or medication allergy. He was safely treated with TAS-102 for the first planned cycle; however, approximately 4 days after receiving the second cycle of TAS-102, he complained of high fever and subsequent dyspnea with severe hypoxemia and went to the emergency room. On examination, his respiratory rate was 33 breaths per minute and fine crackles were heard by chest asuculatation. Laboratory values upon admission were: white blood cell (WBC) count 4100/μL (neutrophils 52.0 %, eosinophils 1.0 %), C-reactive protein (CRP) 6.13 mg/dL (normally under 0.10 mg/mL), lactate dehydrogenase (LDH) 482 IU/mL (normally 114–220 IU/mL), alkaline phosphatase 415 IU/mL (normally 124–367 IU/mL), and KL-6 562 U/mL (normally 105–401 U/mL). Blood gas under oxygen supplementation (10 L/min) revealed a pH of 7.51, pO_2_ of 50.3 mmHg, pCO2 of 34.3 mmHg, and HCO_3_- 27.9 mmol/L. All infectious workup tests were performed, and the results were all negative including common bacteria, fungi, Pneumocystis, Legionella and Cytomegalovirus. A chest radiograph revealed a ground-glass opacity and lower consolidation (Fig. 1). Furthermore, a chest computed tomography (CT) scan showed thickening of the bronchovascular bundles and interlobular septum, diffuse areas of defined ground-glass opacities with fine reticulation and pleural effusions in both lung fields (Fig. 2). Of note, Fig. 2 shows a nodular lesion in the left middle lung field. The appearance of the abnormalities in the CT scan was described as lesions in the form of ground-glass opacities with visible polygonal structures, of the so-called crazy paving pattern. It also revealed a metastatic nodular lesion in the left middle lung field.

**Fig. 3:**
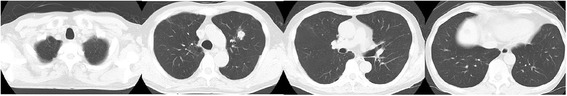
Computed tomography scan approximately 12 days after discontinuing TAS-102 treatment showing improvement of interstitial pneumonitis

The rapid onset and nature of these changes were consistent with drug-induced fibrotic lung disease secondary to TAS-102 chemotherapy. In addition, a peripheral blood drug-induced lymphocyte stimulation test (DLST) with TAS-102 showed that the value for TAS-102 was higher than that of control [973 vs. 476 counts per minute (cpm)]. Thus, he commenced empirical broad-spectrum antibiotics and high-dose intravenous corticosteroid therapy (1000 mg/day for 3 days). Because of his severe dyspnea, bronchoscopy and bronchoalveolar lavage were not performed. Three days later, there was a marked improvement in terms of symptoms and oxygen requirements. Intravenous antibiotics were stopped and oral prednisolone commenced. A CT scan performed approximately 12 days after the prednisolone treatments began showed improvements of the previously observed pulmonary lesions (Fig. 3). Blood gas analysis revealed complete recovery of pulmonary function. Our patient was discharged home with oral prednisolone (20 mg/day). Unfortunately, he died from mCRC disease progression approximately 1 year after the last administration of TAS-102.

## Discussion

Several major breakthroughs, including improvements to standard chemotherapy regimens and molecularly targeted agents, have been achieved in the treatment of mCRC. These advances have increased the survival of mCRC patients in the past 20 years from 12 months to approximately 30 months [8, 9]. This has led to increased numbers of heavily pretreated mCRC patients presenting to daily clinical practices where clinicians have few further treatments to offer. TAS-102 is a new treatment option for patients who have failed all the standard therapies. The reason for this is as follows; TAS-102 is an oral combination drug consisting of trifluridine, which is a thymidine-based nucleoside analog, and tipiracil hydrochloride, which improves the bioavailability of trifluridine by inhibiting its catabolism by thymidine phosphorylase [10]. In the RECOURSE trial, TAS-102 significantly increased overall survival in comparison to placebo, reducing the risk of death more than 30 % in refractory, heavily pretreated mCRC patients. In addition, TAS-102 was found to be a well-tolerated drug in this trial. In the TAS-102 group, only one treatment-related death due to septic shock occurred and only 4 % of patients discontinued treatment because of any toxic effects. In terms of toxicity, TAS-102 is associated with a favorable overall safety profile, but treatment is accompanied by a significant increase of hematological toxicity, in particular 38 % of patients experienced grade 3–4 neutropenia, although febrile neutropenia was observed in only 4 % of patients. Furthermore, the frequency of hematological toxicity was shown to peak during the first cycle and gradually taper off with subsequent cycles.

Incidences of IP associated with TAS-102 have not been reported. Using the key words “TAS-102,” “pneumonitis” and “interstitial” on PubMed identifies no reports of lung toxicity in patients receiving TAS-102. However, IP was noted in seven of 3373 patients from 1209 institutes according to an EPPV on TAS-102 in Japan. Consequently, these EPPV data have noted that the incidence rate of ILD for TAS-102 was comparable to other standard cytotoxic agents. The previous report of ILD in Japanese patients with lung cancer suggested that increased age, poor performance status, reduced normal lung area on CT scans, preexisting chronic ILD, and concurrent cardiac disease were risk factors for ILD [11]. Indeed, our patient had minor emphysematous changes on chest CT upon his cancer diagnosis and a history of heavy smoking. No other reported risk factors for ILD were observed in our patient.

In general, approximately 15–50 % of ILD-related deaths occurred in patients receiving other cytotoxic drugs. Nonetheless, ILD-related deaths associated with TAS-102 chemotherapy were not reported in the previous EPPV. Our results will help clinicians improve ILD outcomes based on prompt cessation of TAS-102 chemotherapy and initiation of corticosteroids. Several types of pulmonary complications associated with oxaliplatin-based treatments have been reported in colorectal cancer patients, including acute lung injury, eosinophilic lung disease, cryptogenic organizing pneumonia and IP or pulmonary fibrosis [12–15]. The exact pathogenesis of TAS-102-induced ILD is unknown. In our case, a positive reaction for TAS-102 was observed upon DLST, and the result suggested a type-1 or type-4 hypersensitivity reaction towards TAS-102. Although new antineoplastic agents are constantly being developed and there are many reports of chemotherapy-induced IP, to the best of our knowledge, this is the first documented case of TAS-102-induced IP.

## Conclusions

In conclusion, we experienced a rare adverse event during TAS-102 chemotherapy. Though TAS-102 is known for its safety and efficacy in clinical practice, clinicians should be especially aware of a possibility of IP associated with TAS-102 therapy; moreover, early diagnosis, careful monitoring and timely management are needed in order to prevent the associated morbidity and mortality.

## Abbreviations

5-Fu: fluorouracil
cpm: count per minute
CEA: carcinoembryonic antigen
CRP: C-reactive protein
CT: computed tomography
DLST: drug-induced lymphocyte stimulation test
EPPV: early post-marketing phase vigilance
ILD: interstitial lung disease
IP: interstitial pneumonia
LDH: lactate dehydrogenase
mCRC: metastatic colorectal cancer
PET/CT: positron emission tomography/computed tomography
WBC: white blood cell
